# Subjektive Gesundheit in der Frühphase der COVID-19-Pandemie – ein Vergleich von soziodemografischen Gruppen und pandemiebezogenen Risikofaktoren

**DOI:** 10.1007/s00103-024-03889-3

**Published:** 2024-05-24

**Authors:** Carolin Heil, Florian Beese, Yong Du, Claudia Hövener, Niels Michalski

**Affiliations:** Abteilung für Epidemiologie und Gesundheitsmonitoring, Robert Koch-Institut, Nordufer 20, 13353 Berlin, Deutschland

**Keywords:** Selbstberichtete Gesundheit, Sozioökonomische Ungleichheit, Vorerkrankungen, Coronavirus, Lockdown, Self-rated health, Socioeconomic inequality, Pre-existing conditions, Coronavirus, Lockdown

## Abstract

**Hintergrund:**

In der Frühphase der COVID-19-Pandemie im Jahr 2020 war der Alltag durch die Eindämmungsmaßnahmen des ersten Lockdowns vergleichsweise stark eingeschränkt, während die SARS-CoV-2-Inzidenzen noch gering ausfielen. Der vorliegende Beitrag analysiert soziodemografische und sozioökonomische Gruppen im Hinblick auf die Beeinträchtigung der subjektiven Gesundheit in dieser Phase.

**Methoden:**

Daten der Hauptbefragung des Sozio-oekonomischen Panels (*n* = 14.856, März–Juli 2020) wurden verwendet, um die relative Häufigkeit selbstberichteter guter Gesundheit, großer Sorgen um die eigene Gesundheit und hoher Lebenszufriedenheit von Männern und Frauen stratifiziert nach Alter, Bildung, Einkommen, Migrationserfahrung, Vorerkrankungen und Risikoberufen zu schätzen. Die Ergebnisse wurden mittels logistischer Regressionen wechselseitig adjustiert und monatsweise dargestellt sowie mit der vorpandemischen Zeit verglichen.

**Ergebnisse:**

Personen in höherem Alter, mit niedriger Bildung oder niedrigem Einkommen sowie mit Vorerkrankungen berichteten seltener positive Gesundheitsoutcomes und häufiger Sorgen. Die Unterschiede zwischen den Merkmalsgruppen blieben im Vergleich zur vorpandemischen Zeit weitgehend stabil. Personen mit niedriger Bildung oder niedrigem Einkommen berichteten im Vergleich zu Personen der mittleren und höheren Bildungs- beziehungsweise Einkommensgruppen zum Zeitpunkt der stärksten Einschränkungen durch Infektionsschutzmaßnahmen seltener eine gute Gesundheit.

**Diskussion:**

Der Einfluss der Frühphase der Pandemie auf die subjektive Gesundheit und Lebenszufriedenheit ist für den Großteil der untersuchten Gruppen gering. Nur für Frauen in niedrigen sozioökonomischen Positionen konnten relative Verschlechterungen identifiziert werden.

**Zusatzmaterial online:**

Zusätzliche Informationen sind in der Online-Version dieses Artikels (10.1007/s00103-024-03889-3) enthalten.

## Abstract (en)

**Background:**

In the early stages of the COVID-19 pandemic in 2020, daily life was significantly restricted due to the containment measures of the initial lockdown while SARS-CoV‑2 incidences remained relatively low. This study analyses socio-demographic and socio-economic groups in terms of changes in their subjective health during this phase.

**Methods:**

Data from the Socio-Economic Panel (*n* = 14,856, March–July 2020) were used to estimate the relative frequency of self-reported good health, great worries about one’s own health, and high life satisfaction of men and women stratified by age, education, income, migration history, pre-existing medical conditions, and high-risk occupation. The results were mutually adjusted using logistic regression, displayed on a monthly basis, and compared with the pre-pandemic period.

**Results:**

Individuals of higher age, with lower education or income, and with pre-existing medical conditions reported positive health outcomes less frequently and worries more often. The differences between the subgroups remained largely stable compared to the pre-pandemic period. During the period of strongest restrictions due to infection-control measures, good health was reported less frequently by individuals with lower education or income compared to individuals with higher education or income.

**Discussion:**

The impact of the early phase of the pandemic on subjective health and life satisfaction was low for the majority of the examined groups. Relative impairments were only identified for women in low socio-economic positions.

## Einleitung

In der Frühphase der COVID-19-Pandemie in Deutschland im März und April 2020 waren die Infektionszahlen, Hospitalisierungen und Sterbefälle aufgrund von SARS-CoV-2(Schweres Akutes Respiratorisches Syndrom Coronavirus 2)-Infektionen noch vergleichsweise gering und regional begrenzt [1]. Verschiedene Studien haben gezeigt, dass die nichtpharmazeutischen Maßnahmen, die ab dem 10.03.2020 eingeleitet wurden, erheblich zur Beherrschung des Infektionsgeschehens beigetragen haben [2, 3]. Gleichzeitig war der Alltag durch diese Eindämmungsmaßnahmen stark eingeschränkt. So wurden Betreuungs- und Bildungsstätten geschlossen, umfassende Kontaktbeschränkungen im öffentlichen und privaten Bereich durchgesetzt sowie weitreichende Schließungen von Dienstleistungsbetrieben veranlasst und öffentliche Veranstaltungen abgesagt [4]. Trotz zunehmender Lockerungen ab dem 20.04. war das Alltagsgeschehen bis in den Sommer 2020 von Einschränkungen geprägt [4]. Durch die Eindämmungsmaßnahmen blieben die Auswirkungen der Pandemie nicht auf die persönliche Betroffenheit durch Infektion begrenzt, sondern betrafen die Gesamtbevölkerung in verschiedensten Lebensbereichen (z. B. Beschäftigung, Familie, gesundheitliche Versorgung) mit Auswirkungen auf den subjektiven Gesundheitszustand [5, 6].

Für die Frühphase der Pandemie liegen zur subjektiven Gesundheit der Bevölkerung in Deutschland vereinzelt Ergebnisse vor, die insgesamt eher eine Verbesserung der subjektiven Gesundheit berichten [7], was auch für weitere europäische Länder gezeigt werden konnte [8–10]. Auch für mit der subjektiven Gesundheit assoziierte Indikatoren wurden im Vergleich des Zeitraums 2015 bis 2019 mit dem Frühjahr 2020 keine Beeinträchtigungen festgestellt, stattdessen wurden eine Reduzierung von Sorgen um die eigene Gesundheit sowie eine stabile Entwicklung der allgemeinen Lebenszufriedenheit in Deutschland berichtet [11]. Offenbar war die Gesundheit der Durchschnittsbevölkerung zunächst nicht wesentlich durch die Pandemie und die Eindämmungsmaßnahmen beeinträchtigt. Andererseits wurde in der sozialepidemiologischen Literatur bereits früh prognostiziert, dass Menschen unterschiedlich stark von den Infektionsschutzmaßnahmen betroffen sein würden und dass ohnehin benachteiligte Personengruppen mit höherer Wahrscheinlichkeit zusätzlich belastet werden würden [12, 13].

Der vorliegende Beitrag unternimmt eine bevölkerungsrepräsentative Deskription sozialer Unterschiede in der subjektiven Gesundheit anhand von Daten der Hauptbefragung des Sozio-oekonomischen Panels (SOEP). Die Größe der Stichprobe lässt einen detaillierten Vergleich von Subgruppen zu, der bisher noch nicht im Hinblick auf die subjektive Gesundheit vorgenommen wurde. Die subjektive Gesundheit wird vor dem Hintergrund der persönlichen Situation (Alter, Gesundheitszustand im Lebensverlauf etc.) eingeschätzt [14], sodass der Indikator erlaubt, die Angaben zwischen Menschen mit unterschiedlichen Lebensumständen zu vergleichen. Daher eignet sich die selbstberichtete Gesundheit dazu, gesundheitliche Ungleichheiten und besonders von der Pandemie betroffene Risikogruppen zu identifizieren. Der selbsteingeschätzte Gesundheitszustand spiegelt nicht nur das Vorliegen von Erkrankungen und Beschwerden wider, sondern beinhaltet auch eine allgemeine Einschätzung des persönlichen Wohlbefindens. Zur Einordnung der Ergebnisse der subjektiven Gesundheit werden außerdem die Sorgen um die eigene Gesundheit und die allgemeine Lebenszufriedenheit als Indikatoren betrachtet. Damit sollen die Ergebnisse zur subjektiven Gesundheit sowohl mit der Beeinträchtigung wahrgenommener gesundheitlicher Bedingungen als auch mit dem allgemeinen Wohlbefinden kontrastiert werden.

Der Beitrag untersucht geschlechtsspezifische Gruppenunterschiede dieser 3 Indikatoren entlang von 6 ausgewählten soziodemografischen und sozioökonomischen Merkmalen. Hintergrund sind wesentliche Unterschiede in der subjektiven Gesundheit nach Alters‑, Bildungs- sowie Einkommensgruppen und der Krankengeschichte, die für den vorpandemischen Zeitraum bekannt sind [15–17]. Zusätzlich werden die Migrationserfahrung sowie die Ausübung eines Risikoberufs betrachtet. Die gewählten Merkmale differenzieren dabei Gruppen mit unterschiedlichen Risiken und Vulnerabilitäten. So zeigen sich Bevölkerungsgruppen unterschiedlich anfällig gegenüber Infektion oder gegenüber Engpässen in der medizinischen Versorgung (Personen höheren Alters, Personen mit Vorerkrankungen; [18]). Bildung und Einkommen werden betrachtet, weil sozioökonomische Ressourcen in vielerlei Hinsicht mit Bedingungen von Gesundheit und Krankheit zusammenhängen (z. B. finanzielle und berufliche Sorgen, Wohnbedingungen, Informations- und Präventionsverhalten; [12, 19]). Personen mit eigener bzw. (groß-)elterlicher Migrationserfahrung werden denen ohne Migrationserfahrung gegenübergestellt, weil sich hier häufig verschiedene Benachteiligungen überlagern (Diskriminierung, Bildungsbenachteiligung, Armutsbetroffenheit, prekäre Beschäftigungsverhältnisse; [20]). Zudem werden erwerbstätige Personen in systemrelevanten Berufen mit Personen in anderen Berufen verglichen, weil sich darin unterschiedliche Infektionsrisiken und zusätzliche Belastungen während des Lockdowns widerspiegeln [21].

Im ersten Schritt der Analyse werden geschlechtsspezifische Unterschiede der subjektiven Gesundheit für verschiedene Bevölkerungsgruppen in der Frühphase der Pandemie 2020 dargestellt und multivariat adjustiert. In einem zweiten Schritt werden die gruppenspezifischen Niveaus (aus Schritt 1) mit dem Mittel der Vorjahre (2015–2019) verglichen. In einem dritten Schritt sollen die monatlichen Trends betrachtet werden. Dabei werden die Gruppen über die Monate März bis Juli 2020 verglichen, wodurch der Zeitraum der höchsten Belastung während der Frühphase der Pandemie besonders in den Blick genommen werden kann.

## Methodik

### Datengrundlage

Die Datengrundlage für die Analysen bildet das SOEP – eine jährlich durchgeführte repräsentative longitudinale Befragung von Privathaushalten in Deutschland, die Analysen zur Soziodemografie und -ökonomie, Gesundheit sowie anderen Themen auf Individualebene ermöglicht [22]. Detailliertere Informationen zum SOEP, zur Stichprobenzusammensetzung und zur Panelstabilität liegen an anderer Stelle vor [23]. Für die Analysen wurden Daten der Haupterhebung des SOEP aus dem Befragungsjahr 2020 von Personen im Alter von 18 Jahren und älter verwendet, die in die Kernfeldzeit der Befragung (März bis Juli 2020) fielen (Frauen: *N* = 7723, Männer *N* = 7133). Der gewählte Zeitraum entspricht der ersten Welle der COVID-19-Pandemie zuzüglich des ersten Teils des darauffolgenden Sommerplateaus (Phasen 1 und 2a) gemäß der Phaseneinteilung des Robert Koch-Instituts [4]. Für den Vergleich mit den vorangegangenen Jahren wurden Daten der gleichen monatlichen Zeiträume aus den Befragungsjahren 2015 bis 2019 hinzugezogen (Frauen: *N* = 39.815, Männer *N* = 35.861).

### Indikatoren

Für die Messung des gegenwärtigen Gesundheitszustands, erhoben mit der Frage: „Wie würden Sie Ihren gegenwärtigen Gesundheitszustand beschreiben?“, werden die Antwortkategorien „sehr gut“ und „gut“ zusammengefasst (gute Gesundheit) und der Zusammenführung von „zufriedenstellend“, „weniger gut“ und „schlecht“ gegenübergestellt.

Sorgen um die eigene Gesundheit wurden mittels der Antwortkategorien „große Sorgen“, „einige Sorgen“ und „keine Sorgen“ erhoben und für die Analysen in „große Sorgen“ und „einige/keine Sorgen“ zusammengefasst.

Die Lebenszufriedenheit wurde auf einer Skala von 0 („ganz und gar unzufrieden“) bis 10 („voll und ganz zufrieden“) abgefragt und für diese Analysen in hohe (Skalenwerte 7–10) und niedrige oder mittlere Lebenszufriedenheit (Skalenwerte 0–6) dichotomisiert.

Für die Messung der Bildung wurde die CASMIN(Comparative Analysis of Social Mobility in Industrial Nations; [24])-Klassifikation in niedrige, mittlere und hohe Bildung unterteilt.

Die Einkommensposition wird an den Kategorien „< 60 %“, „60–150 %“ und „150 % und mehr“ des empirischen Medians des Nettoäquivalenzeinkommens gemessen.

Das Vorliegen mindestens einer Vorerkrankung wurde als „Ja, mind. eine“ kategorisiert, wenn mindestens eine Vorerkrankung vorlag, die als Risikofaktor für einen schweren COVID-19-Verlauf gilt oder wo Beeinträchtigungen durch pandemiebedingte Einschränkungen erwartet werden können (Diabetes mellitus, Asthma bronchiale, Herzkrankheit, Krebserkrankung, Schlaganfall, Migräne, Bluthochdruck, depressive Erkrankung, Demenzerkrankung, Gelenkerkrankung; [18, 25]).

Zur Operationalisierung des Migrationsstatus wurde die vom SOEP aufbereitete Variable verwendet, die zwischen „keiner“, „eigener“ und „(groß-)elterlicher Migrationserfahrung“ unterscheidet.

Für die Zuordnung von Berufen mit besonderem Ansteckungsrisiko wurden die Angaben zum aktuellen Beruf der Befragten genutzt [26].

Als zusätzliche Kontrollvariablen wurden der Familienstatus und die Erwerbstätigkeit in die multivariaten Analysen aufgenommen. Eine detailliertere Beschreibung der gewählten Indikatoren befindet sich im Onlinematerial.

### Statistische Auswertungen

Im ersten Schritt wurden Prävalenzen der Endpunkte von März bis Juli für die Beobachtungszeiträume 2020 und 2015 bis 19 geschätzt. Die Gruppenunterschiede der Prävalenzen für das Jahr 2020 wurden in multivariaten logistischen Regressionsmodellen wechselseitig und unter Hinzunahme des Familienstandes und der Erwerbstätigenstatus adjustiert. In einem zweiten Schritt wurden die adjustierten Prävalenzen beider Beobachtungszeiträume grafisch dargestellt. Im dritten Schritt wurden die Regressionsmodelle für den Zeitraum 2020 jeweils um Interaktionseffekte zwischen den Stratifizierungsmerkmalen und dem Untersuchungsmonat ergänzt. Basierend auf diesen Modellen wurden durchschnittliche vorhergesagte monatliche Prävalenzen (März bis Juli) für alle Ausprägungen der Stratifizierungsmerkmale berechnet und grafisch dargestellt, wobei die Schätzer für Juni und Juli aufgrund geringer Fallzahlen in diesen Monaten zusammengeführt wurden.

Alle Berechnungen wurden für Frauen und Männer getrennt durchgeführt. Für die Analyse nach Risiko- und Nichtrisikoberufen wurde die Stichprobe auf die Population beschränkt, die eine Erwerbstätigkeit berichtet und sich im erwerbstätigen Alter befindet (18–65 Jahre). Dadurch wurde gewährleistet, dass Personen in Risikoberufen mit erwerbstätigen Personen in anderen Berufen verglichen werden konnten. Sämtliche Analysen wurden gewichtet durchgeführt, um das Stichprobendesign zu berücksichtigen, selektive Ausfallwahrscheinlichkeiten zu korrigieren und bezüglich zentraler demografischer Merkmale an die Verteilung des Mikrozensus anzupassen. Für die korrekte Berechnung der Standardfehler wurde die Clusterung in Haushalten berücksichtigt. Tab. 1 zeigt die Zusammensetzung der Stichprobe.

**Tab. 1:** Stichprobenzusammensetzung stratifiziert nach Geschlecht

		Frauen (*N* = 7723)		Männer (*N* = 7133)		Gesamt (*N* = 14.856)	
		*n*	%^a^	*n*	%^a^	*n*	%^a^
Alter	18–24	632	7,54	587	8,25	1219	7,89
	25–39	1679	24,09	1431	25,66	3110	24,86
	40–54	2397	25,24	2151	25,82	4548	25,53
	55–69	2052	25,23	1940	25,72	3992	25,47
	70+	962	17,85	1024	14,54	1986	16,22
	Keine Angabe	1	0,05	0	0,00	1	0,03
Bildung	Niedrig	1798	29,04	1878	30,39	3676	29,71
	Mittel	3526	43,98	2701	37,70	6227	40,88
	Hoch	2171	23,60	2296	27,66	4467	25,60
	Keine Angabe	228	3,38	258	4,25	486	3,81
Einkommen	< 60 %	1298	17,55	968	14,06	2266	15,83
	60–150 %	4710	65,75	4157	64,07	8867	64,92
	≥ 150 %	1695	16,67	1990	21,86	3685	19,23
	Keine Angabe	20	0,03	18	0,01	38	0,02
Migrationserfahrung	Keine	5640	75,49	5342	75,25	10.982	75,37
	Eigene	1473	18,26	1217	17,20	2690	17,74
	(Groß‑)Elterliche	610	6,26	574	7,55	1184	6,89
	Keine Angabe	0	0	0	0	0	0
Familienstatus	Verheiratet, zusammenlebend	4450	51,62	4423	54,00	8873	52,79
	Verheiratet, dauernd getrennt lebend	183	2,10	127	2,23	310	2,16
	Ledig	1780	26,15	1931	33,63	3711	29,84
	Geschieden	788	10,29	473	7,34	1261	8,83
	Verwitwet	489	9,28	158	2,55	647	5,96
	Keine Angabe	33	0,57	21	0,25	54	0,41
Erwerbstätigenstatus	Nicht erwerbstätig	2785	39,51	2055	31,11	4840	35,37
	Erwerbstätig	4938	60,49	5078	68,89	10.016	64,63
	Keine Angabe	0	0	0	0	0	0
Risikoberuf^b^	Nein	2898	36,90	3595	52,98	6493	44,82
	Ja	1131	14,21	292	4,47	1423	9,41
	Keine Angabe	761	7,51	934	8,81	1695	8,15
	Nicht erwerbstätig/> 65 Jahre	2933	41,39	2312	33,74	5245	37,62
Vorerkrankungen	Keine	3358	41,24	3177	44,87	6535	43,03
	Ja, mind. eine	3674	49,80	3314	45,08	6988	47,47
	Keine Angabe	691	8,96	642	10,06	1333	9,50
Befragungsmonat	März	3250	44,24	2847	42,14	6097	43,20
	April	2472	31,48	2289	32,42	4761	31,94
	Mai	1327	17,33	1313	17,78	2640	17,55
	Juni/Juli	674	6,96	684	7,67	1358	7,31
Gegenwärtiger Gesundheitszustand	Schlecht, weniger gut, zufriedenstellend	3624	51,33	3029	45,22	6653	48,32
	Gut, sehr gut	4087	48,46	4089	54,65	8176	51,51
	Keine Angabe	12	0,20	15	0,14	27	0,17
Sorgen um die eigene Gesundheit	Keine, einige	6487	83,10	6251	86,24	12.738	84,65
	Große	1213	16,59	856	13,46	2069	15,05
	Keine Angabe	23	0,31	26	0,30	49	0,30
Allgemeine Lebenszufriedenheit	Niedrig	1357	19,67	1191	19,37	2548	19,52
	Hoch	6347	80,04	5926	80,44	12.273	80,24
	Keine Angabe	19	0,29	16	0,19	35	0,24

^a^gewichtete Prozentangaben

^b^Berufe mit besonderem Ansteckungsrisiko

## Ergebnisse

### Bi- und multivariate Ergebnisse

Tab. 2 zeigt die geschlechtsstratifizierten Prävalenzschätzungen der zentralen Indikatoren subjektiver Gesundheit nach soziodemografischen und sozioökonomischen Gruppen für das Jahr 2020. Insgesamt berichtete etwa die Hälfte der Personen eine gute Gesundheit. Etwa 15,5 % der Frauen und 12,9 % der Männer machten sich große Sorgen um die eigene Gesundheit. Der Anteil hoher Lebenszufriedenheit lag bei beiden Geschlechtern bei jeweils 80,6 %.

**Tab. 2:** Geschätzte Prävalenzen und 95 %-Konfidenzintervalle (KI) für zentrale Indikatoren subjektiver Gesundheit nach Risikogruppen 2020

		Gesundheit „Gut–Sehr gut“				Große Sorge um die eigene Gesundheit				Anteil hohe Lebenszufriedenheit			
Altersgruppe	*–*	*Frauen N* *=* *7710*		*Männer N* *=* *7118*		*Frauen N* *=* *7699*		*Männer N* *=* *7107*		*Frauen N* *=* *7703*		*Männer N* *=* *7117*	
		*%*	*KI (95* *%)*	*%*	*KI (95* *%)*	*%*	*KI (95* *%)*	*%*	*KI (95* *%)*	*%*	*KI (95* *%)*	*%*	*KI (95* *%)*
	18–24 Jahre	76,5	[70,95–81,29]	80,1	[74,05–85,04]	6,8	[4,62–9,75]	6,8	[4,43–10,33]	85,2	[80,32–89,00]	82,7	[77,52–86,84]
	25–39 Jahre	68,9	[65,26–72,27]	72,6	[68,77–76,02]	9,2	[7,25–11,63]	8,8	[6,69–11,59]	84,7	[81,80–87,27]	82,4	[78,97–85,33]
	40–54 Jahre	52,5	[49,09–55,90]	56,0	[52,27–59,58]	15,8	[13,41–18,43]	13,8	[11,30–16,63]	81,4	[78,47–83,96]	80,9	[77,72–83,68]
	55–69 Jahre	36,4	[33,31–39,55]	43,1	[39,64–46,55]	20,9	[18,23–23,75]	18,0	[15,38–20,95]	77,5	[74,48–80,20]	78,5	[75,43–81,25]
	70+	20,9	[17,77–24,36]	27,3	[23,46–31,51]	26,2	[22,46–30,35]	17,2	[14,01–20,91]	74,5	[70,21–78,34]	79,5	[75,08–83,30]
	Total	48,2	[46,26–50,08]	55,3	[53,27–57,26]	15,5	[14,25–16,88]	12,9	[11,63–14,33]	80,6	[79,05–81,96]	80,6	[79,06–82,14]
Bildung (CASMIN)	–	*Frauen N* *=* *7487*		*Männer N* *=* *6862*		*Frauen N* *=* *7476*		*Männer N* *=* *6851*		*Frauen N* *=* *7478*		*Männer N* *=* *6861*	
		*%*	*KI (95* *%)*	*%*	*KI (95* *%)*	*%*	*KI (95* *%)*	*%*	*KI (95* *%)*	*%*	*KI (95* *%)*	*%*	*KI (95* *%)*
	Niedrig	33,5	[30,18–36,96]	41,0	[37,69–44,4]	28,1	[25,17–31,30]	21,2	[18,47–24,12]	73,5	[70,14–76,51]	73,8	[70,63–76,81]
	Mittel	49,4	[46,72–52,00]	58,6	[55,60–61,52]	13,7	[12,07–15,53]	11,7	[9,87–13,80]	80,9	[78,69–82,88]	82,0	[79,53–84,27]
	Hoch	63,1	[59,57–66,41]	62,5	[59,09–65,80]	8,7	[6,86–10,92]	7,4	[5,83–9,46]	87,3	[84,79–89,41]	86,5	[83,94–88,62]
Einkommensposition	–	*Frauen N* *=* *7691*		*Männer N* *=* *7100*		*Frauen N* *=* *7680*		*Männer N* *=* *7090*		*Frauen N* *=* *7684*		*Männer N* *=* *7099*	
		*%*	*KI (95* *%)*	*%*	*KI (95* *%)*	*%*	*KI (95* *%)*	*%*	*KI (95* *%)*	*%*	*KI (95* *%)*	*%*	*KI (95* *%)*
	< 60 %	38,7	[34,38–43,24]	49,3	[44,37–54,21]	25,5	[22,09–29,18]	23,5	[19,46–28,00]	69,8	[65,51–73,80]	67,7	[62,75–72,34]
	60–150 %	48,3	[46,10–50,53]	53,3	[50,97–55,59]	15,9	[14,41–17,58]	13,9	[12,29–15,62]	81,1	[79,26–82,76]	80,6	[78,60–82,47]
	150 % und mehr	59,9	[55,58–64,07]	62,5	[58,57–66,18]	10,3	[7,75–13,57]	6,02	[4,39–8,21]	88,2	[85,04–90,74]	88,8	[86,21–91,02]
Vorerkrankungen	–	*Frauen N* *=* *7024*		*Männer N* *=* *6478*		*Frauen N* *=* *7016*		*Männer N* *=* *6470*		*Frauen N* *=* *7016*		*Männer N* *=* *6477*	
		*%*	*KI (95* *%)*	*%*	*KI (95* *%)*	*%*	*KI (95* *%)*	*%*	*KI (95* *%)*	*%*	*KI (95* *%)*	*%*	*KI (95* *%)*
	Keine	68,3	[65,57–70,88]	70,7	[68,00–73,31]	7,4	[6,13–8,93]	7,9	[6,41–9,66]	86,3	[84,23–88,12]	86,4	[84,28–88,30]
	Ja, mind. eine	31,8	[29,62–34,14]	37,5	[34,97–40,09]	24,2	[22,04–26,39]	19,6	[17,54–21,93]	75,1	[72,78–77,31]	74,2	[71,68–76,63]
Migrationserfahrung	–	*Frauen N* *=* *7711*		*Männer N* *=* *7118*		*Frauen N* *=* *7700*		*Männer N* *=* *7107*		*Frauen N* *=* *7704*		*Männer N* *=* *7117*	
		*%*	*KI (95* *%)*	*%*	*KI (95* *%)*	*%*	*KI (95* *%)*	*%*	*KI (95* *%)*	*%*	*KI (95* *%)*	*%*	*KI (95* *%)*
	Keine	48,2	[46,21–50,26]	53,1	[51,03–55,16]	15,6	[14,22–17,13]	11,7	[10,44–13,12]	80,1	[78,37–81,64]	81,0	[79,29–82,64]
	Eigene	44,3	[39,64–49,02]	54,1	[49,09–58,97]	23,0	[19,48–26,92]	21,8	[18,05–26,16]	79,7	[75,58–83,27]	78,6	[74,09–82,56]
	(Groß‑)Elterliche	65,0	[58,17–71,33]	72,3	[65,32–78,35]	10,6	[7,33–14,96]	12,4	[7,75–19,27]	84,6	[79,17–88,76]	80,8	[73,66–86,33]
Risikoberuf	–	*Frauen N* *=* *4036*		*Männer N* *=* *3898*		*Frauen N* *=* *4028*		*Männer N* *=* *3895*		*Frauen N* *=* *4032*		*Männer N* *=* *3901*	
		%	*KI (95* *%)*	*%*	*KI (95* *%)*	*%*	*KI (95* *%)*	*%*	*KI (95* *%)*	*%*	*KI (95* *%)*	*%*	*KI (95* *%)*
	Nein	60,2	[57,25–63,01]	62,8	[60,22–65,33]	10,7	[8,97–12,60]	9,9	[8,43–11,55]	84,1	[81,83–86,15]	84,4	[82,43–86,21]
	Ja	51,9	[46,94–56,82]	67,6	[59,53–74,69]	12,4	[9,83–15,63]	8,6	[5,16–13,85]	79,9	[75,68–83,56]	84,0	[77,42–88,90]
	Total	57,9	[55,37–60,39]	63,2	[60,74–65,59]	11,1	[9,68–12,75]	9,8	[8,40–11,34]	83,0	[81,00–84,86]	84,4	[82,49–86,10]

Gewichtete Ergebnisse

*N* ungewichtet

Mit zunehmendem Alter wurde seltener eine gute bis sehr gute Gesundheit, häufiger große Sorgen um die Gesundheit und seltener eine hohe Lebenszufriedenheit berichtet. In Gruppen mit hohen Bildungsabschlüssen waren die Prävalenzen einer gut oder sehr gut berichteten Gesundheit und der hohen Lebenszufriedenheit höher und die Häufigkeit großer Sorgen geringer als unter Befragten mit niedriger Bildung. Die Unterschiede für die subjektive Gesundheit fielen bei den Frauen deutlicher aus als bei Männern. Ähnliche Befunde zeigten sich auch beim Einkommen. Deutliche Unterschiede zeigten sich zwischen Befragten mit und ohne Vorerkrankungen. Personen ohne Vorerkrankungen berichteten etwa doppelt so häufig eine sehr gute oder gute Gesundheit, etwa 3‑mal so häufig große Sorgen und seltener hohe Lebenszufriedenheit als Personen mit mindestens einer Vorerkrankung. In Hinblick auf die Migrationserfahrung berichteten Personen mit (groß-)elterlicher Migrationserfahrung häufiger eine gute bis sehr gute Gesundheit als Personen ohne oder mit eigener Migrationserfahrung. Personen mit eigener Migrationserfahrung berichteten häufiger große Sorgen als Personen ohne Migrationserfahrung. Während Frauen in Risikoberufen seltener eine gute bis sehr gute Gesundheit berichteten als Frauen in anderen Berufen, ließen sich bezüglich der Sorgen um die Gesundheit und des Anteils hoher Lebenszufriedenheit keine Unterschiede entlang dieses Merkmals erkennen.

Die meisten Subgruppenunterschiede blieben unter wechselseitiger Adjustierung und Berücksichtigung weiterer Merkmale bestehen. Für die Sorgen um die Gesundheit lag unter wechselseitiger Kontrolle allerdings kein Altersgradient mehr vor. Der Anteil großer Sorgen war für Männer und Frauen der mittleren Altersgruppe (40–54 Jahre) am größten, alle anderen Gruppen unterschieden sich nicht signifikant. Des Weiteren veränderten sich die Unterschiede in der subjektiven Gesundheit nach Migrationserfahrung bei den Frauen. Frauen mit Migrationserfahrung wiesen signifikant geringere Chancen für gute Gesundheit auf als Frauen ohne Migrationserfahrung. Die Unterschiede im selbstberichteten Gesundheitszustand waren zwischen Frauen in Risikoberufen und Frauen in anderen Berufen nach Adjustierung nicht mehr nachweisbar (Tabelle A1–2 im Onlinematerial).

Beim Vergleich mit den Jahren 2015 bis 2019 zeigten sich in fast allen Subgruppen ähnliche Tendenzen: Gute beziehungsweise sehr gute Gesundheit sowie hohe Lebenszufriedenheit wurden im Jahr 2020 häufiger und große Sorgen seltener berichtet (Abbildung A1, Tabelle A3 im Onlinematerial). Dabei fielen die Unterschiede in den meisten Subgruppen gering aus. Einzelne Gruppen brachen allerdings aus dem Muster der Tendenz für eine Verbesserung der Gesundheit aus. So wiesen die Prävalenzschätzungen für die ältesten Personen sowie für Frauen in der niedrigen Bildungs- und Einkommensgruppe keinen Anstieg in der Prävalenz guter Gesundheit auf. Zudem sank der Anteil der Frauen mit großen Sorgen vergleichsweise schwach. Frauen mit hohem Einkommen machten sich häufiger große Sorgen um ihre Gesundheit. Bei der hohen Lebenszufriedenheit wies die jüngste Altersgruppe der Männer keinen Anstieg im Anteil hoher Lebenszufriedenheit auf.

### Trends der subjektiven Gesundheit in der Frühphase der Pandemie

**Abb. 1:**
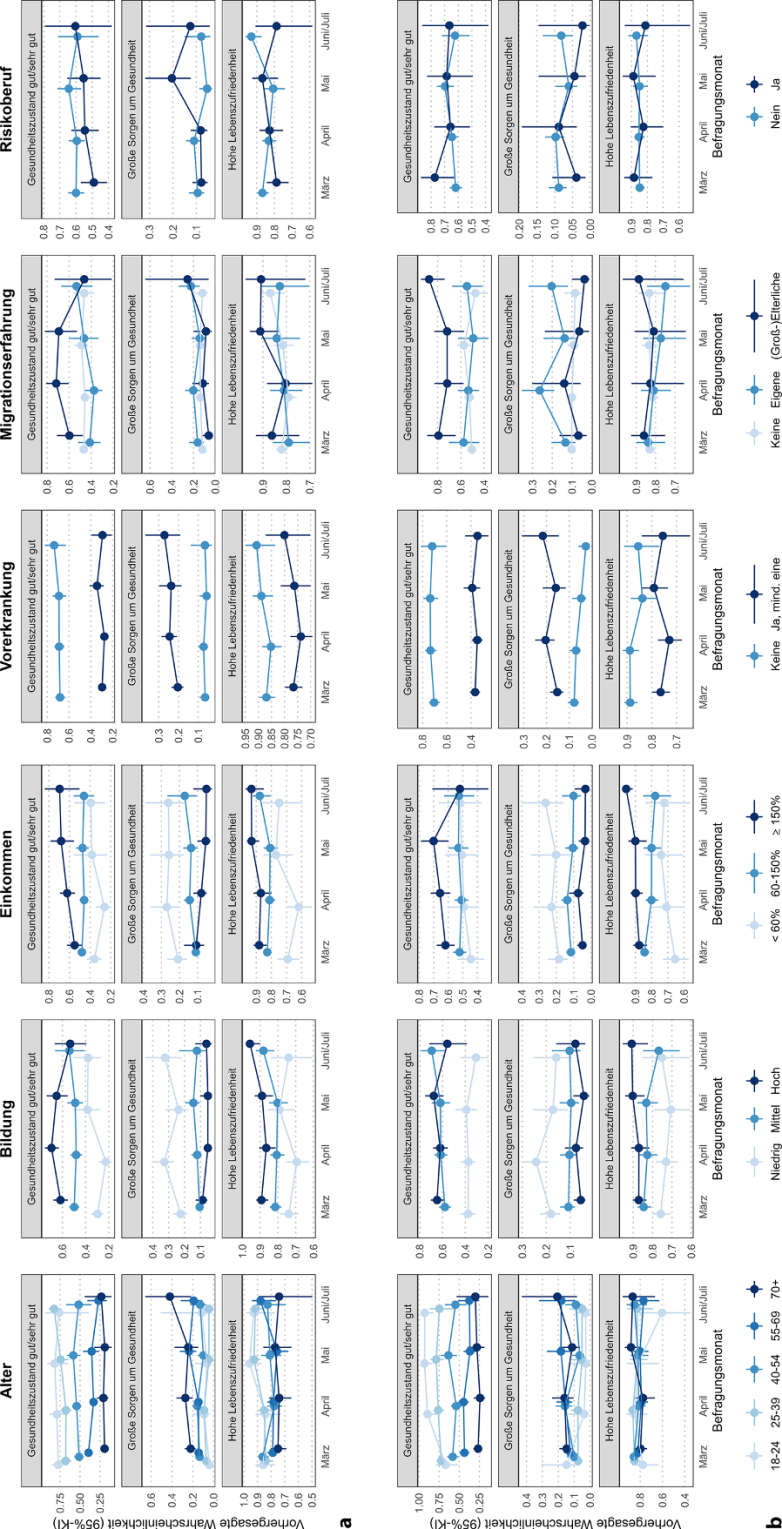
Trends der subjektiven Gesundheit nach Stratifizierungsmerkmalen von März bis Juni/Juli 2020 für Frauen (**a**) und Männer (**b**). (Die Schätzungen für Juni und Juli sind aufgrund geringer Fallzahlen in den einzelnen Monaten zusammengefasst. Hinweis: Für eine pointierte Darstellung der Unterschiede variieren die *y‑Achsen* in der Skalierung.) *KI* Konfidenzintervall. (Quelle: eigene Abbildung)

Abb. 1 stellt monatliche Trends (März bis Juli 2020) der vorhergesagten Wahrscheinlichkeiten für Frauen (A) und Männer (B) für die betrachteten Risikogruppen dar. Während die Grafiken erneut die wesentlichen Gruppenunterschiede nach Alter, Bildung, Einkommen und Personen mit mind. einer und ohne Vorerkrankungen herausstellen, ließen sich für bestimmte Gruppen in einzelnen Monaten Abweichungen identifizieren.

So zeigte sich für Frauen, dass sich die Unterschiede in der subjektiven Gesundheit und bei den Sorgen um die Gesundheit nach Bildung von März bis April 2020 vergrößerten und ab Mai wieder anglichen. Frauen mit geringer Bildung berichteten im April seltener eine gute Gesundheit und machten sich signifikant häufiger große Sorgen als im März. In den Folgemonaten erhöhten sich die Anteile guter subjektiver Gesundheit bei Frauen mit niedriger Bildung, der Anteil großer Sorgen ging nicht signifikant zurück. Für Männer mit niedriger Bildung ließ sich ein ähnlicher Ausschlag eines höheren Anteils großer Sorgen im April ausmachen, wobei die Unterschiede zwischen den Monaten nicht signifikant waren.

Besonderheiten im April 2020 zeigten sich auch für alle 3 Outcomes für Frauen mit niedrigem Einkommen: Die Anteile guter subjektiver Gesundheit und hoher Lebenszufriedenheit lagen im April jeweils niedriger als im März und in den Folgemonaten. Der Anteil großer Sorgen war in dieser Gruppe ab April leicht erhöht. Bei Männern waren in Bezug auf die Einkommensgruppen keine deutlichen Abweichungen in den Trends zu erkennen.

Die Unterschiede nach Vorerkrankungen, die bereits im gesamten Querschnitt am größten ausfielen, waren auch in allen Monaten für alle Outcomes deutlich zu erkennen. Bei der Betrachtung großer Sorgen ließ sich feststellen, dass sich die Unterschiede zwischen Personen mit mindestens einer Vorerkrankungen leicht vergrößerten. Bei Männern war dafür nicht nur ein leichter Anstieg im Anteil großer Sorgen bei Personen mit mindestens einer Vorerkrankungen verantwortlich, sondern auch ein Rückgang der Anteile großer Sorgen bei Personen ohne Vorerkrankungen.

Entlang des Merkmals Migrationserfahrung zeigten Frauen beim gegenwärtigen Gesundheitszustand von März bis April zunächst steigende Ungleichheiten, wobei Frauen mit (groß-)elterlicher Migrationserfahrung häufiger einen guten bis sehr guten Gesundheitszustand berichteten als Frauen mit eigener und ohne Migrationserfahrung. Ab Mai glichen sich die Trends wieder an. Zudem zeigten Männer mit eigener Migrationserfahrung häufiger große Sorgen um die eigene Gesundheit im April als Männer ohne Migrationserfahrung. Unter Männern mit eigener und (groß-)elterlicher Migrationserfahrung fiel ein erhöhter Anteil großer Sorgen im April gegenüber März auf, der sich in den Folgemonaten wieder einebnete. Aufgrund von vergleichsweise geringen Fallzahlen in den Gruppen mit Migrationserfahrung waren die Schätzungen allerdings mit größerer Unsicherheit behaftet. Bei der Betrachtung nach Risikoberufen lässt sich allenfalls im Mai ein größerer Anteil von Frauen in Risikoberufen erkennen, die große Sorgen um die eigene Gesundheit berichteten.

## Diskussion

### Zusammenfassung

Der vorliegende Beitrag hat Unterschiede zwischen verschiedenen soziodemografischen und sozioökonomischen Gruppen für 2 subjektive Indikatoren der Gesundheit und einem Indikator der Lebensqualität in der Frühphase der Pandemie analysiert. Die Unterschiede in der subjektiven Gesundheit wurden im Wesentlichen durch das Alter und die Krankengeschichte, aber auch durch die sozioökonomische Position und die Migrationserfahrung geprägt. Im Vergleich des Pandemiejahres 2020 mit dem Mittel der 5 Vorjahre haben sich die Unterschiede kaum verändert. So zeigten sich auch in der vorliegenden Analyse, dass die Anteile guter subjektiver Gesundheit und hoher Lebenszufriedenheit für die Mehrheit der Subgruppen minimal angestiegen waren. Gleichzeitig war der Anteil der Personen, die große Sorgen berichteten, in den meisten Subgruppen leicht zurückgegangen. Eine Ausnahme stellten die Frauen der höheren Einkommensgruppe dar, die im Vergleich zu den vorpandemischen Jahren größere Sorgen berichteten. Der Blick auf die monatliche Entwicklung zeigte die höchsten Anteile großer Sorgen in dieser Gruppe im März 2020. Möglicherweise spielt hier eine Rolle, dass das Infektionsgeschehen der ersten Welle in sozioökonomisch besser gestellten Bevölkerungsgruppen und in den wohlhabenden Regionen Süddeutschlands seinen Ausgang nahm [1]. Eine insgesamt tendenzielle Verbesserung der subjektiv berichteten Gesundheit und des Wohlbefindens wurde auch in anderen Studien mit verwandten Outcomes der mentalen Gesundheit und gesundheitsbezogenen Lebensqualität gefunden [7, 11]. Eine Erklärung hierfür könnte sein, dass es in Reaktion auf den noch neuartigen Erreger SARS-CoV‑2 zunächst zu größerer Solidarität und stärkerem gesellschaftlichen Zusammenhalt für die gemeinsame Krisenbewältigung kam [27–29], die auch Auswirkungen auf die subjektive Gesundheit hatten. Ein weiterer möglicher Grund ist, dass Personen in der Pandemie eher Vergleiche mit den medial kolportierten Bildern von schwer an COVID-19 erkrankten Personen zogen, was den Referenzzustand für die Einschätzung der eigenen Gesundheit im Vergleich zur vorpandemischen Zeit verändert haben könnte [11]. Das gilt insbesondere, da SARS-CoV-2-Infektionen, Hospitalisierungen und Sterbefälle im Vergleich zu späteren Wellen vergleichsweise gering waren, sich auf einzelne Regionen beschränkten und persönliche Betroffenheit durch COVID-19 deshalb noch nicht so verbreitet war. Selbst in Gruppen, für die bereits früh ein erhöhtes Risiko für schwere Verläufe und Mortalität bekannt war, insbesondere Personen in höherem Lebensalter und mit bestimmten Vorerkrankungen, zeigte sich keine erhöhte Häufigkeit großer Sorgen [30, 31].

Insgesamt spiegeln sich in den Subgruppenunterschieden die Erkenntnisse aus der vorpandemischen Zeit wider, in denen neben Alter und chronischen sowie akuten Erkrankungen auch sozioökonomische Unterschiede eine wesentliche Rolle spielten, die zum Beispiel im Rahmen der Gesundheitsberichterstattung dokumentiert sind [15]. Die bekannten Ungleichheiten der subjektiven Gesundheit zwischen Bildungsgruppen werden in der Literatur über die Ausübung von Berufen mit schlechteren Arbeitsbedingungen, geringere psychosoziale Ressourcen zur Problembewältigung oder mit risikobehaftetem Gesundheitsverhalten erklärt [32]. Die vorgelegten Analysen zeigen für alle 3 untersuchten Outcomes auch nach gegenseitiger Adjustierung nicht nur Bildungs-, sondern auch Einkommensunterschiede. Ein stabiler Einfluss des Einkommens auf die subjektive Gesundheit wurde für die Erwerbsbevölkerung vor und nach der Pandemie in anderen Studien aufgezeigt [33]. Finne und Razum bestätigen in ihrer Analyse, dass das Einkommen der bedeutendste Faktor für die individuelle Veränderung subjektiver Gesundheit in der Frühphase der Pandemie ist [34].

Die Ergebnisse zeigen andererseits einzelne Ausnahmen aus dem Muster gleichbleibender Unterschiede. Für Personen in der höchsten Altersgruppe, bei Befragten mit eigener Migrationserfahrung und bei Frauen mit niedrigem Einkommen wurde im Vergleich zum vorpandemischen Zeitraum ein leichter Rückgang des Anteils von Personen mit guter subjektiver Gesundheit beobachtet. In der Betrachtung der monatlichen Trends für 2020 wurde deutlich, dass sich die Befunde, die auf eine Verschlechterung der Gesundheit in einzelnen Subgruppen hindeuten, vor allem im April 2020 häuften – dem Monat, in dem die höchsten Inzidenzen, Hospitalisierungen und Todesfälle in Deutschland zu verzeichnen waren und in denen die Infektionsschutzmaßnahmen am stärksten in den Alltag eingriffen [4]. Die monatliche Betrachtung zeigte entsprechend, dass Frauen in den niedrigen Bildungsgruppen im April seltener gute Gesundheit berichteten und sich ab April häufiger große Sorgen machten. Ähnliche Muster deuten sich auch bei Frauen der niedrigen Einkommensgruppe und für Männer mit niedriger Bildung an. Diese Effekte könnten darauf zurückgeführt werden, dass Personen der niedrigen Einkommens- und Bildungsgruppe aufgrund ihrer Erwerbstätigkeit nicht die Möglichkeit hatten, im Homeoffice zu arbeiten und somit einem erhöhten Infektionsrisiko ausgesetzt waren oder auch Ungewissheit gegenüberstanden [35, 36].

Dass Frauen in niedrigen sozioökonomischen Positionen von den Auswirkungen der Pandemie besonders betroffen sein würden, wurde schon früh prognostiziert [37]. Nach der Schließung von Kitas und Schulen übernahmen meist die Frauen die Kinderbetreuung [38, 39]. Sie waren damit häufiger der Doppelbelastung von Betreuungs- und Erwerbsarbeit ausgesetzt [37]. Besonders alleinerziehende Eltern (in großer Mehrheit Frauen) und Eltern aus der niedrigen Bildungsgruppe berichteten über stärkere Belastungen während des Lockdowns [40]. Auch wenn diese verstärkten Mehrfachbelastungen von Müttern in der COVID-19-Pandemie hier nicht direkt analysiert wurden, korrespondieren die festgestellten geringeren Anteile hoher Lebenszufriedenheit und guter subjektiver Gesundheit sowie größerer Sorgen unter Frauen mit niedriger Bildung und niedrigem Einkommen mit dem Befund besonderer Risiken für Frauen. Ein weiteres Indiz für diese Interpretation ist, dass sich Frauen im mittleren Erwerbsalter (40–54 Jahre) nach Kontrolle von Vorerkrankungen, Bildung, Einkommen und Erwerbsstatus häufiger große Sorgen um die Gesundheit machten als Frauen in anderen Altersgruppen. Auch hier ist zu vermuten, dass der höhere familiäre Organisationsaufwand und Unterstützungsbedarf in der Betreuungsarbeit nach Schulschließungen häufig von Frauen in dieser Altersgruppe getragen wurde und häufiger mit großen Sorgen um die Gesundheit verbunden war. Zudem arbeiten Frauen häufiger in Berufen, die sowohl systemrelevant sind als auch häufig schlechter bezahlt werden als andere Berufe [41], was die Prekarität weiter verschärft haben dürfte.

Bezogen auf die Migrationserfahrung zeigen unsere Ergebnisse beim subjektiven Gesundheitszustand zunächst höhere Prävalenzen bei Personen mit (groß-)elterlicher Migrationserfahrung. Diese gleichen sich mit der Adjustierung aus, was sich mit einem potenziell jüngeren Alter der Personen in dieser Gruppe erklären lässt. Personen mit eigener Migrationserfahrung wiesen eine höhere Prävalenz großer Sorgen um die eigene Gesundheit auf – ein Befund, der sich auch nach multivariater Adjustierung als robust zeigte und Gegenstand weiterer Analysen sein sollte.

### Stärken und Limitationen

Die vorliegende Analyse verwendete Daten der SOEP-Hauptbefragung, die für geschlechtergetrennte Auswertungen im Untersuchungszeitraum eine ausreichend hohe Fallzahl bot und so auch ermöglichte, kleine Unterschiede zwischen soziodemografischen und sozioökonomischen Gruppen bevölkerungsrepräsentativ zu identifizieren. Die Verwendung der Daten der SOEP-Hauptbefragung erlaubte den Rückgriff auf Daten, die mit gewohnten Befragungsinstrumenten im regulären Zyklus erhoben wurden, sodass themenbedingte Priming-Effekte vermindert werden konnten. Die Vergleichbarkeit der Querschnitte mit dem vorpandemischen Kontext wurde außerdem durch die Betrachtung gleicher Beobachtungszeiträume aufrechterhalten. Weiterhin wurden systematische Panelausfälle im SOEP und die Clusterung der Fälle in Haushalten durch komplexe Surveygewichtung berücksichtigt. Auf Basis hoher Fallzahlen konnten die geschlechtergetrennten Subgruppenunterschiede auch monatsweise analysiert werden, wenn auch mit zum Teil weiten Konfidenzintervallen, sodass Veränderungen konkreter auf den unmittelbaren Einfluss der Pandemiebedingungen zurückgeführt werden konnten. Dies ermöglichte ein differenziertes Bild, das in dieser Form bisher für Deutschland noch nicht vorlag. Demgegenüber sind jedoch auch Limitation zu nennen. Durch den Pandemiebeginn im Frühjahr 2020 sind Veränderungen des Antwortverhaltens aufgrund abweichender Befragungssituationen, wie dem Wechsel der Interviewmethode zu Telefoninterviews, nicht auszuschließen. Die Geflüchteten-Stichproben sowie Migrations-Aufstockungsstichproben wurden ausgeschlossen, weil die Feldphase im Jahr 2020 für diese Stichproben erst im Juli begann.^1^ Lediglich für die Monate Juni/Juli, für die eine geringe Fallzahl vorlag, wich die Zusammensetzung der Stichrobe bezüglich der Teilstichproben des SOEP leicht von der Gesamtstichprobe ab. Aus Kapazitätsgründen wurde für die Analysen nur eine Auswahl soziodemografischer und sozioökonomischer Merkmale berücksichtigt. Weitere Gruppen, wie beispielsweise Alleinerziehende, oder regionale Unterschiede blieben unberücksichtigt und bedürfen weiterer Forschung. In zukünftigen Untersuchungen sollte auch die intersektionale Perspektive, also das Zusammenwirken benachteiligender Merkmale, stärker berücksichtigt werden.

### Fazit

Trotz einer Vielzahl von Belastungen und Unsicherheiten, die im Frühjahr 2020 durch den Beginn der COVID-19-Pandemie entstanden sind, war die subjektive Gesundheit der Befragten verhältnismäßig wenig beeinträchtigt. Gute subjektive Gesundheit war in bekannten Risikogruppen zwar auch zu Beginn der Pandemie seltener verbreitet, die Risikogruppen fielen aber auch nicht deutlich ab. Im Detail zeigen die Ergebnisse, dass bestimmte Personengruppen von der Pandemie und den Eindämmungsmaßnahmen besonders betroffen waren. Diese Befunde sind für die Betrachtung gesundheitlicher Ungleichheit in pandemischen Krisenzeiten relevant und sollten für differenzierte Analysen von Präventionspotenzialen genutzt werden.

### Supplementary Information

Beschreibung der Indikatoren und Stratifizierungsmerkmale

## Footnotes

1 Weil sich die Stichprobe des SOEP aus verschiedenen Teilstichproben zusammensetzt, die bestimmte Bevölkerungsgruppen (z. B. Ostdeutsche, migrierte und geflüchtete Personen sowie Hocheinkommensbeziehende) überproportional einschließen und Panelausfälle ausgleichen (Auffrischungs- und Ergänzungsstichproben; [23]), wurde geprüft, ob sich die Anteile der Teilstichproben der analysierten Stichprobe für die betrachteten Monate unterscheidet.
