# Supplementary material for: Subjective health in the early phase of the COVID-19 pandemic—a comparison of socio-demographic groups and pandemic-related risk factors
Source: Bundesgesundheitsblatt Gesundheitsforschung Gesundheitsschutz. 2024 May 24;67(7):843–53. [Article in German] doi: 10.1007/s00103-024-03889-3 (PMC11231003; doi:10.1007/s00103-024-03889-3)
Supplement: Supplementary file 1 — Beschreibung der Indikatoren und Stratifizierungsmerkmale [file 103_2024_3889_MOESM1_ESM.docx]

Onlinematerial

Subjektive Gesundheit in der Frühphase der COVID-19-Pandemie – ein Vergleich von soziodemografischen Gruppen und pandemiebezogenen Risikofaktoren

Carolin Heil^1^, Florian Beese^1^, Yong Du^1^, Claudia Hövener^1^, Niels Michalski^1^

^1^ Abteilung für Epidemiologie und Gesundheitsmonitoring, Robert Koch-Institut

Beschreibung der Indikatoren und Stratifizierungsmerkmale

Selbsteingeschätzter Gesundheitszustand

Der selbsteingeschätzte Gesundheitszustand wurde mit der Frage „Wie würden Sie Ihren gegenwärtigen Gesundheitszustand beschreiben?“ erhoben und konnte mit den Antwortkategorien „sehr gut“, „gut“, „zufriedenstellend“, „weniger gut“ und „schlecht“ beantwortet werden.

Sorgen um die eigene Gesundheit

Sorgen wurden im Sozio-oekonomischen Panel (SOEP) in Bezug auf verschiedene Bereiche in einer Itembatterie mit der Frage „Wie ist es mit den folgenden Gebieten – machen Sie sich da Sorgen?“ erhoben. Für die vorliegenden Analysen wurde das Item „um die eigene Gesundheit“ mit den Antwortkategorien „große Sorgen“, „einige Sorgen“ und „keine Sorgen“ zur Abbildung der Sorgen um die eigene Gesundheit verwendet.

Allgemeine Lebenszufriedenheit

Die allgemeine Lebenszufriedenheit wurde mit der Frage „Wie zufrieden sind Sie gegenwärtig, alles in allem, mit Ihrem Leben?“ erhoben. Die befragten Personen können sich auf einer Skala von 0 („ganz und gar unzufrieden“) bis 10 („voll und ganz zufrieden“) einordnen. Die allgemeine Lebenszufriedenheit mit einem Cut-Off-Skalenwert zwischen 6 und 7 dichotomisiert. Dieser Cut-Off-Wert grenzt die vier höchsten Werte der elfstufigen Skala von den sieben niedrigeren Werten ab. Insgesamt beträgt der Anteil hoher Lebenszufriedenheit etwa 80% (vs. 20% geringe Lebenszufriedenheit). Dieses Prävalenzverhältnis ist dazu geeignet, unter den gegebenen Fallzahlen bedeutungsvolle Differenzen statistisch abzusichern.

Weitere Stratifizierungsindikatoren

Zur Identifizierung von Unterschieden in der subjektiven Gesundheit wurden neben Alter und Geschlecht verschiedene Stratifizierungsmerkmale für die Analysen herangezogen: Der Bildungsstatus wurde mithilfe der neunstufigen Comparative Analysis of Social Mobility in Industrial Nations- (CASMIN-)Klassifizierung erfasst und zu den Kategorien „niedrig“, „mittel“ und „hoch“ zusammengefasst.

Die Einkommensangaben beziehen sich auf das Nettoäquivalenzeinkommen des Vorjahres inkl. Wohneigentum.

Vorerkrankungen wurden durch die Frage „Hat ein Arzt bei Ihnen jemals eine oder mehrere der folgenden Krankheiten festgestellt?“ erfragt, die in einer Liste der häufigsten Erkrankungen und mit den Antwortmöglichkeiten „sonstige Krankheit“ sowie „keine Krankheit festgestellt“ beantwortet werden konnte. Für die vorliegenden Analysen wurde eine Variable mit den Ausprägungen „keine“ und „ja, mindestens eine“ bekannte Vorerkrankung gebildet (Vorerkrankungen werden im SOEP alle zwei Jahre mit in den Fragebogen aufgenommen).

Die Operationalisierung des Migrationsstatus erfolgte mittels der im SOEP vorliegenden Variable zur Migrationserfahrung, die nach keiner, direkter und indirekter Migrationserfahrung unterscheidet. Keine Migrationserfahrung liegt vor, wenn die befragte Person sowie beide Elternteile in Deutschland geboren wurden. Eine direkte (eigene) Migrationserfahrung liegt vor, wenn die befragte Person nicht in Deutschland geboren wurde. Eine indirekte ((groß-)elterliche) Migrationserfahrung liegt vor, wenn mindestens ein Elternteil und im Falle von fehlenden Informationen der Eltern mindestens ein Großelternteil nicht in Deutschland geboren wurde.

Als weiteres Stratifizierungsmerkmal wurde die Ausübung eines Risikoberufs mit den Ausprägungen „kein Risikoberuf“ und „Risikoberuf“ genutzt. Im SOEP werden die Berufe anhand der Klassifizierung der Berufe 2010 (KldB 2010^[[1]](#footnote-1)^) erfasst. Grundlage für die Zuordnung der Berufe in Risiko- bzw. Nicht-Risikoberufe ist eine Klassifikation für systemrelevante Berufe des Instituts für Arbeitsmarkt- und Berufsforschung. Darunter zählen „Arzt und Praxishilfe“, „Gesundheits- und Krankpflege, Rettungsdienst, Geburtenhilfe“, „Human- und Zahnmedizin“, „Altenpflege“, „Erziehung, Sozialarbeit, Heilerziehungspflege“, „Verkauf von Lebensmitteln“, „Verkauf von drogerie- und apothekenüblichen Waren, Sanitäts- und Medizinbedarf“, „Polizeivollzugs- und Kriminaldienst, Gerichts- und Justizvollzug“ und „Reinigung“^[[2]](#footnote-2)^.

Als zusätzliche Kontrollvariablen der Soziodemografie dienen für die durchgeführten Analysen die Variablen des Familienstandes sowie des Erwerbsstatus. Für den Familienstand zusammengefasst wurden die im SOEP vorgegebene Variable des Familienstandes in die Kategorien „verheiratet, mit Ehepartner zusammenlebend“, „verheiratet, dauernd getrennt lebend“, „ledig", „geschieden/eingetragene gleichgeschlechtliche Partnerschaft aufgehoben“, „verwitwet/Lebenspartner/-in aus eingetragener gleichgeschlechtlicher Partnerschaft verstorben“. Der Erwerbsstatus wurde durch die im SOEP vorliegende Variable aufgenommen und dichotomisiert in die Kategorien „nicht erwerbstätig“ und „erwerbstätig“.

**Tabelle A1** Ergebnisse der multivariaten logistischen Regression für die gewählten Indikatoren für Frauen und Männer (März – Juli 2020)

|  | **Gegenwärtiger Gesundheitszustand** | | **Sorgen um die Gesundheit** | | **Lebenszufriedenheit** | |
| --- | --- | --- | --- | --- | --- | --- |
|  | **Frauen N=6804** | **Männer N=6232** | **Frauen N=6795** | **Männer N=6225** | **Frauen N=6793** | **Männer N=6231** |
|  | OR  [95%-KI] | OR  [95%-KI] | OR  [95%-KI] | OR  [95%-KI] | OR  [95%-KI] | OR  [95%-KI] |
| **Altersgruppe** (Ref.: 40-54 Jahre) | | | | | | |
| 18-24 Jahre | 3,571*** | 3,580*** | 0,406** | 0,541 | 2,433*** | 0,926 |
|  | [2,250-5,669] | [2,114-6,063] | [0,207-0,796] | [0,243-1,200] | [1,438-4,115] | [0,546-1,570] |
| 25-39 Jahre | 1,826*** | 1,997*** | 0,570** | 0,655 | 1,426* | 1,114 |
|  | [1,420-2,347] | [1,474-2,705] | [0,396-0,822] | [0,420-1,020] | [1,048-1,941] | [0,780-1,592] |
| 55-69 Jahre | 0,685*** | 0,848 | 0,757 | 0,906 | 0,964 | 1,166 |
|  | [0,550-0,854] | [0,662-1,087] | [0,565-1,016] | [0,627-1,308] | [0,726-1,280] | [0,855-1,590] |
| 70+ | 0,446*** | 0,706 | 0,613* | 0,494** | 1,106 | 1,807** |
|  | [0,317-0,629] | [0,491-1,016] | [0,416-0,903] | [0,311-0,786] | [0,744-1,643] | [1,172-2,786] |
|  |  |  |  |  |  |  |
| **Bildung** (Ref.: Niedrig) | | | | | | |
| Mittel | 1,122 | 1,616*** | 0,607*** | 0,720* | 1,177 | 1,492** |
|  | [0,905-1,390] | [1,289-2,025] | [0,469-0,786] | [0,540-0,958] | [0,925-1,499] | [1,145-1,944] |
| Hoch | 1,733*** | 1,961*** | 0,396*** | 0,457*** | 1,863*** | 1,809*** |
|  | [1,354-2,220] | [1,519-2,532] | [0,284-0,552] | [0,321-0,650] | [1,362-2,549] | [1,331-2,459] |
|  |  |  |  |  |  |  |
| **Einkommensposition** (Ref.: <60%) | | | | | | |
| 60-150% | 1,377* | 0,968 | 0,684** | 0,804 | 1,737*** | 1,370* |
|  | [1,062-1,786] | [0,731-1,282] | [0,522-0,896] | [0,565-1,145] | [1,334-2,263] | [1,013-1,854] |
| 150% u. m. | 1,803*** | 1,332 | 0,631* | 0,466** | 2,418*** | 2,037*** |
|  | [1,303-2,494] | [0,957-1,853] | [0,403-0,985] | [0,289-0,754] | [1,614-3,623] | [1,374-3,019] |
|  |  |  |  |  |  |  |
| **Erwerbsstatus** (Ref.: nicht erwerbstätig) | | | | | | |
| Erwerbstätig | 1,271* | 1,775*** | 0,540*** | 0,464*** | 1,181 | 1,761*** |
|  | [1,034-1,563] | [1,364-2,311] | [0,419-0,696] | [0,328-0,657] | [0,910-1,532] | [1,332-2,328] |
|  |  |  |  |  |  |  |
| **Familienstand** (Ref.: Verheiratet, mit Ehepartner zusammend lebend) | | | | | | |
| Verheiratet, getrennt lebend | 1,011  [0,565-1,810] | 1,285  [0,676-2,445] | 1,493  [0,707-3,155] | 0,946  [0,439-2,039] | 0,556^*^  [0,324-0,953] | 0,592  [0,285-1,231] |
|  |  |  |  |  |  |  |
| Ledig | 0,770^*^ | 0,851 | 1,163 | 1,170 | 0,642^**^ | 0,691^*^ |
|  | [0,597-0,994] | [0,649-1,118] | [0,814-1,660] | [0,791-1,731] | [0,478-0,860] | [0,511-0,935] |
| Geschieden | 0,673^**^ | 1,053 | 1,186 | 1,609^*^ | 0,752 | 0,558^**^ |
|  | [0,511-0,887] | [0,763-1,454] | [0,861-1,633] | [1,088-2,381] | [0,557-1,016] | [0,385-0,809] |
| Verwitwet | 0,880 | 0,962 | 1,347 | 1,788 | 0,775 | 1,095 |
|  | [0,627-1,236] | [0,576-1,607] | [0,941-1,927] | [0,973-3,287] | [0,541-1,113] | [0,601-1,995] |
|  |  |  |  |  |  |  |
| **Migrationserfahrung** (Ref.: Keine Migrationserfahrung) | | | | | | |
| Eigene | 0,742^*^ | 1,086 | 1,398^*^ | 1,812^***^ | 1,052 | 1,024 |
|  | [0,574-0,959] | [0,815-1,447] | [1,045-1,871] | [1,308-2,511] | [0,774-1,429] | [0,732-1,432] |
| (Groß-) Elterliche | 0,954  [0,678-1,341] | 1,633^*^  [1,079-2,471] | 1,057  [0,644-1,736] | 0,964  [0,500-1,856] | 1,117  [0,721-1,732] | 1,160  [0,707-1,902] |
|  |  |  |  |  |  |  |
|  |  |  |  |  |  |  |
| **Vorerkrankung** (Ref.: Trifft nicht zu) | | | | | | |
| Ja | 0,348^***^ | 0,385^***^ | 2,898^***^ | 2,397^***^ | 0,607^***^ | 0,469^***^ |
|  | [0,293-0,414] | [0,318-0,466] | [2,228-3,769] | [1,805-3,183] | [0,484-0,760] | [0,365-0,603] |
| **Beobachtungen** | 6.804 | 6.232 | 6.795 | 6.225 | 6.793 | 6.231 |

Gewichtete Ergebnisse; OR = Odds Ration, KI = Konfidenzintervall, Ref. = Referenz, u. m. = und mehr; * p < 0,05, ** p < 0,01, *** p < 0,001

**Tabelle A2** Ergebnisse der multivariaten logistischen Regression für die gewählten Indikatoren für Frauen und Männer in der erwerbstätigen Bevölkerung zwischen 18 und 65 Jahren (März – Juli 2020)

|  | **Gegenwärtiger Gesundheitszustand** | | **Sorgen um die Gesundheit** | | **Lebenszufriedenheit** | |
| --- | --- | --- | --- | --- | --- | --- |
|  | **Frauen** | **Männer** | **Frauen** | **Männer** | **Frauen** | **Männer** |
|  | OR  [95%-KI] | OR  [95%-KI] | OR  [95%-KI] | OR  [95%-KI] | OR  [95%-KI] | OR  [95%-KI] |
| **Altersgruppe** (Ref.: 40-54 Jahre) | | | | | | |
| 18-24 Jahre | 2,934^***^ | 4,539^***^ | 0,497 | 0,824 | 2,145^*^ | 1,118 |
|  | [1,548-5,559] | [2,276-9,053] | [0,196-1,255] | [0,312-2,177] | [1,042-4,416] | [0,577-2,167] |
| 25-39 Jahre | 1,633^**^ | 2,128^***^ | 0,702 | 0,676 | 1,060 | 1,175 |
|  | [1,217-2,190] | [1,532-2,957] | [0,442-1,114] | [0,403-1,136] | [0,741-1,517] | [0,788-1,753] |
| 55-65 Jahre | 0,621^***^ | 0,778 | 0,837 | 1,040 | 0,780 | 1,171 |
|  | [0,476-0,810] | [0,587-1,031] | [0,569-1,230] | [0,668-1,620] | [0,551-1,103] | [0,809-1,694] |
|  |  |  |  |  |  |  |
| **Bildung** (Ref.: Niedrig) | | | | | | |
| Mittel | 0,908 | 1,805^***^ | 0,520^**^ | 0,803 | 1,157 | 1,310 |
|  | [0,664-1,244] | [1,341-2,430] | [0,345-0,784] | [0,528-1,222] | [0,796-1,681] | [0,905-1,895] |
| Hoch | 1,359 | 1,907^***^ | 0,263^***^ | 0,467^**^ | 1,909^**^ | 1,704^*^ |
|  | [0,953-1,939] | [1,368-2,658] | [0,159-0,437] | [0,275-0,791] | [1,230-2,964] | [1,109-2,616] |
|  |  |  |  |  |  |  |
| **Einkommensposition** (Ref.: <60%) | | | | | | |
| 60-150% | 1,790^**^ | 0,677 | 0,863 | 0,582 | 1,873^**^ | 0,985 |
|  | [1,205-2,658] | [0,448-1,021] | [0,533-1,397] | [0,332-1,021] | [1,228-2,857] | [0,588-1,649] |
| 150% u. m. | 2,043^**^ | 0,974 | 0,887 | 0,364^**^ | 2,596^**^ | 1,502 |
|  | [1,286-3,246] | [0,607-1,563] | [0,436-1,804] | [0,183-0,724] | [1,414-4,767] | [0,819-2,755] |
|  |  |  |  |  |  |  |
| **Familienstand** (Ref.: Verheiratet, mit Ehepartner zusammend lebend) | | | | | | |
| Verheiratet, getrennt lebend | 0,930  [0,463-1,870] | 1,136  [0,496-2,600] | 1,414  [0,507-3,939] | 1,324  [0,493-3,555] | 0,609  [0,307-1,207] | 0,522  [0,193-1,413] |
|  |  |  |  |  |  |  |
| Ledig | 0,878 | 0,821 | 1,377 | 1,047 | 0,755 | 0,645^*^ |
|  | [0,650-1,186] | [0,604-1,116] | [0,876-2,163] | [0,637-1,721] | [0,531-1,073] | [0,450-0,925] |
| Geschieden | 0,662^*^ | 0,900 | 1,161 | 1,592 | 0,850 | 0,609 |
|  | [0,467-0,937] | [0,610-1,327] | [0,709-1,899] | [0,881-2,877] | [0,552-1,308] | [0,359-1,035] |
| Verwitwet | 1,488 | 3,321 | 0,600 | 0,0844^*^ | 0,821 | 1,652 |
|  | [0,704-3,145] | [0,902-12,22] | [0,217-1,657] | [0,010-0,712] | [0,323-2,083] | [0,302-9,033] |
|  |  |  |  |  |  |  |
| **Migrationserfahrung** (Ref.: Keine Migrationserfahrung) | | | | | | |
| Eigene | 0,647^**^ | 1,085 | 1,472 | 1,620^*^ | 1,150 | 1,255 |
|  | [0,467-0,897] | [0,774-1,521] | [0,972-2,229] | [1,045-2,512] | [0,768-1,724] | [0,814-1,933] |
| (Groß-) Elterliche | 0,958  [0,635-1,443] | 1,686^*^  [1,049-2,711] | 1,131  [0,624-2,050] | 0,869  [0,369-2,045] | 1,171  [0,682-2,011] | 0,807  [0,477-1,365] |
|  |  |  |  |  |  |  |
|  |  |  |  |  |  |  |
| **Vorerkrankung** (Ref.: Trifft nicht zu) | | | | | | |
| Ja | 0,368^***^ | 0,434^***^ | 2,388^***^ | 2,360^***^ | 0,731^*^ | 0,491^***^ |
|  | [0,296-0,457] | [0,345-0,547] | [1,715-3,325] | [1,646-3,384] | [0,546-0,979] | [0,361-0,667] |
|  |  |  |  |  |  |  |
| **Risikoberuf** (Ref.: Kein Risikoberuf) | | | | | | |
| Ja | 0,905 | 1,204 | 0,893 | 0,615 | 0,958 | 1,048 |
|  | [0,708-1,157] | [0,803-1,805] | [0,630-1,266] | [0,325-1,166] | [0,703-1,306] | [0,650-1,689] |
| **Beobachtungen** | 3.845 | 3.659 | 3.837 | 3.657 | 3.840 | 3.661 |

Gewichtete Ergebnisse; OR = Odds Ration, KI = Konfidenzintervall, Ref. = Referenz, u. m. = und mehr; * p < 0,05, ** p < 0,01, *** p < 0,001

**Tabelle A3** Prävalenzschätzungen und 95%-Konfidenzintervalle für zentrale Indikatoren subjektiver Gesundheit für Frauen und Männer nach Risikogruppen 2015-2019

|  |  | **Gesundheit "Gut-Sehr gut"** | | | | **"Große" Sorge um die eigene Gesundheit** | | | | **Anteil hohe Lebenszufriedenheit** | | | | |
| --- | --- | --- | --- | --- | --- | --- | --- | --- | --- | --- | --- | --- | --- | --- |
| **Altersgruppe** |  | **Frauen N=39.774** | | **Männer N=35.829** | | **Frauen N=39.707** | | **Männer N=35.773** | | **Frauen N=39.743** | | **Männer N=35.786** | | |
|  |  | % | KI (95%) | % | KI (95%) | % | KI (95%) | % | KI (95%) | % | KI (95%) | % | KI (95%) |  |
|  | 18-24 Jahre | 73,0 | [69,99-75,84] | 80,7 | [78,30-82,94] | 12,7 | [10,85-14,88] | 9,6 | [8,00-11,54] | 83,04 | [80,20-85,54] | 83,89 | [81,25-86,21] |  |
|  | 25-39 Jahre | 66,0 | [64,01-67,89] | 69,9 | [67,74-72,03] | 12,5 | [11,26-13,82] | 10,5 | [9,17-11,98] | 83,03 | [81,32-84,60] | 81,58 | [79,69-83,33] |  |
|  | 40-54 Jahre | 48,7 | [46,76-50,72] | 51,3 | [49,17-53,49] | 17,6 | [16,28-19,09] | 16,1 | [14,57-17,71] | 78,78 | [77,17-80,30] | 76,55 | [74,63-78,37] |  |
|  | 55-69 Jahre | 36,3 | [34,27-38,30] | 37,9 | [35,85-40,03] | 23,1 | [21,23-25,00] | 19,2 | [17,59-20,98] | 73,65 | [71,70-75,51] | 75,76 | [73,76-77,66] |  |
|  | 70+ | 21,9 | [19,89-23,98] | 28,9 | [26,45-31,43] | 29,3 | [27,06-31,56] | 23,1 | [20,85-25,55] | 69,96 | [67,59-72,22] | 75,92 | [73,51-78,17] |  |
|  | Total | 46,0 | [44,84-47,09] | 52,4 | [51,17-53,58] | 18,9 | [18,07-19,71] | 15,4 | [14,55-16,22] | 77,54 | [76,61-78,44] | 78,31 | [77,32-79,27] |  |
| **Bildung (CASMIN)** |  | **Frauen N=38.655** | | **Männer N=34.638** | | **Frauen N=38.591** | | **Männer N=34.589** | | **Frauen N=38.625** | | **Männer N=34.599** | | |
|  |  | % | KI (95%) | % | KI (95%) | % | KI (95%) | % | KI (95%) | % | KI (95%) | % | KI (95%) |  |
|  | Niedrig | 33,5 | [31,73-35,35] | 39,8 | [37,92-41,61] | 28,9 | [27,25-30,57] | 24,9 | [23,25-26,55] | 70,1 | [68,36-71,83] | 71,5 | [69,61-73,23] |  |
|  | Mittel | 49,1 | [47,50-50,77] | 55,8 | [53,78-57,73] | 17,4 | [16,19-18,72] | 13,0 | [11,83-14,35] | 78,7 | [77,39-80,02] | 79,6 | [77,94-81,15] |  |
|  | Hoch | 58,5 | [56,19-60,70] | 59,6 | [57,30-61,82] | 11,0 | [9,78-12,24] | 9,2 | [8,07-10,48] | 84,5 | [82,78-86,05] | 85,1 | [83,58-86,54] |  |
| **Einkommens-position** |  | **Frauen N=39.745** | | **Männer N=35.782** | | **Frauen N=39.679** | | **Männer N=35.726** | | **Frauen N=39.715** | | **Männer N=35.738** | | |
|  |  | % | KI (95%) | % | KI (95%) | % | KI (95%) | % | KI (95%) | % | KI (95%) | % | KI (95%) |  |
|  | <60% | 39,0 | [36,71-41,27] | 45,8 | [43,10-48,48] | 30,8 | [28,65-33,03] | 26,6 | [24,22-29,18] | 64,6 | [62,35-66,80] | 63,1 | [60,41-65,77] |  |
|  | 60-150% | 46,2 | [44,88-47,47] | 51,5 | [50,12-52,88] | 19,4 | [18,46-20,43] | 15,8 | [14,85-16,79] | 78,0 | [76,85-79,01] | 78,8 | [77,67-79,96] |  |
|  | 150% und mehr | 54,7 | [52,18-57,26] | 57,6 | [55,16-60,08] | 9,5 | [8,22-10,83] | 8,6 | [7,47-9,79] | 86,3 | [84,64-87,78] | 87,1 | [85,48-88,52] |  |
| **Vorerkrankungen** |  | **Frauen N=37.265** | | **Männer N=33.588** | | **Frauen N=37.202** | | **Männer N=33.535** | | **Frauen N=37.239** | | **Männer N=33.551** | | |
|  |  | % | KI (95%) | % | KI (95%) | % | KI (95%) | % | KI (95%) | % | KI (95%) | % | KI (95%) |  |
|  | Keine | 65,1 | [63,66-66,58] | 67,8 | [66,39-69,25] | 10,6 | [9,82-11,45] | 9,1 | [8,31-9,97] | 83,9 | [82,66-84,97] | 82,7 | [81,39-83,90] |  |
|  | Ja, mindestens eine | 30,4 | [29,09-31,65] | 33,8 | [32,33-35,38] | 27,1 | [25,80-28,44] | 23,6 | [22,27-25,05] | 72,2 | [70,77-73,50] | 73,0 | [71,38-74,59] |  |
| **Migrations-erfahrung** |  | **Frauen N=39.774** | | **Männer N=35.829** | | **Frauen N=39.707** | | **Männer N=35.773** | | **Frauen N=39.743** | | **Männer N=35.786** | | |
|  |  | % | KI (95%) | % | KI (95%) | % | KI (95%) | % | KI (95%) | % | KI (95%) | % | KI (95%) |  |
|  | Keine | 44,6 | [43,29-45,81] | 49,7 | [48,38-51,10] | 18,7 | [17,74-19,68] | 14,6 | [13,68-15,50] | 76,8 | [75,66-77,82] | 78,4 | [77,21-79,44] |  |
|  | Eigene | 48,7 | [46,14-51,27] | 55,4 | [52,58-58,16] | 25,3 | [23,26-27,36] | 22,6 | [20,41-24,95] | 77,6 | [75,53-79,52] | 77,0 | [74,47-79,33] |  |
|  | (Groß-)Elterliche | 62,8 | [58,79-66,73] | 67,3 | [63,37-70,96] | 12,9 | [10,83-15,39] | 13,9 | [11,49-16,77] | 80,8 | [77,51-83,78] | 79,0 | [75,47-82,21] |  |
| **Risikoberuf** |  | **Frauen N=22.809** | | **Männer N=22.609** | | **Frauen N=22.773** | | **Männer N=22.574** | | **Frauen N=22.788** | | **Männer N=22.585** | | |
|  |  | % | KI (95%) | % | KI (95%) | % | KI (95%) | % | KI (95%) | % | KI (95%) | % | KI (95%) |  |
|  | Nein | 56,8 | [55,13-58,43] | 59,2 | [57,71-60,65] | 12,1 | [11,14-13,21] | 11,4 | [10,60-12,30] | 82,9 | [81,70-84,10] | 82,3 | [81,15-83,43] |  |
|  | Ja | 51,3 | [48,73-53,89] | 59,9 | [54,55-65,09] | 17,1 | [15,46-18,89] | 11,8 | [9,09-15,05] | 78,6 | [76,24-80,78] | 78,8 | [74,35-82,62] |  |
|  | Total | 55,3 | [53,90-56,73] | 59,3 | [57,81-60,66] | 13,3 | [12,45-14,28] | 11,5 | [10,65-12,30] | 81,8 | [80,73-82,90] | 82,1 | [80,94-83,15] |  |

Gewichtete Ergebnisse; N ungewichtet; KI=Konfidenzintervall


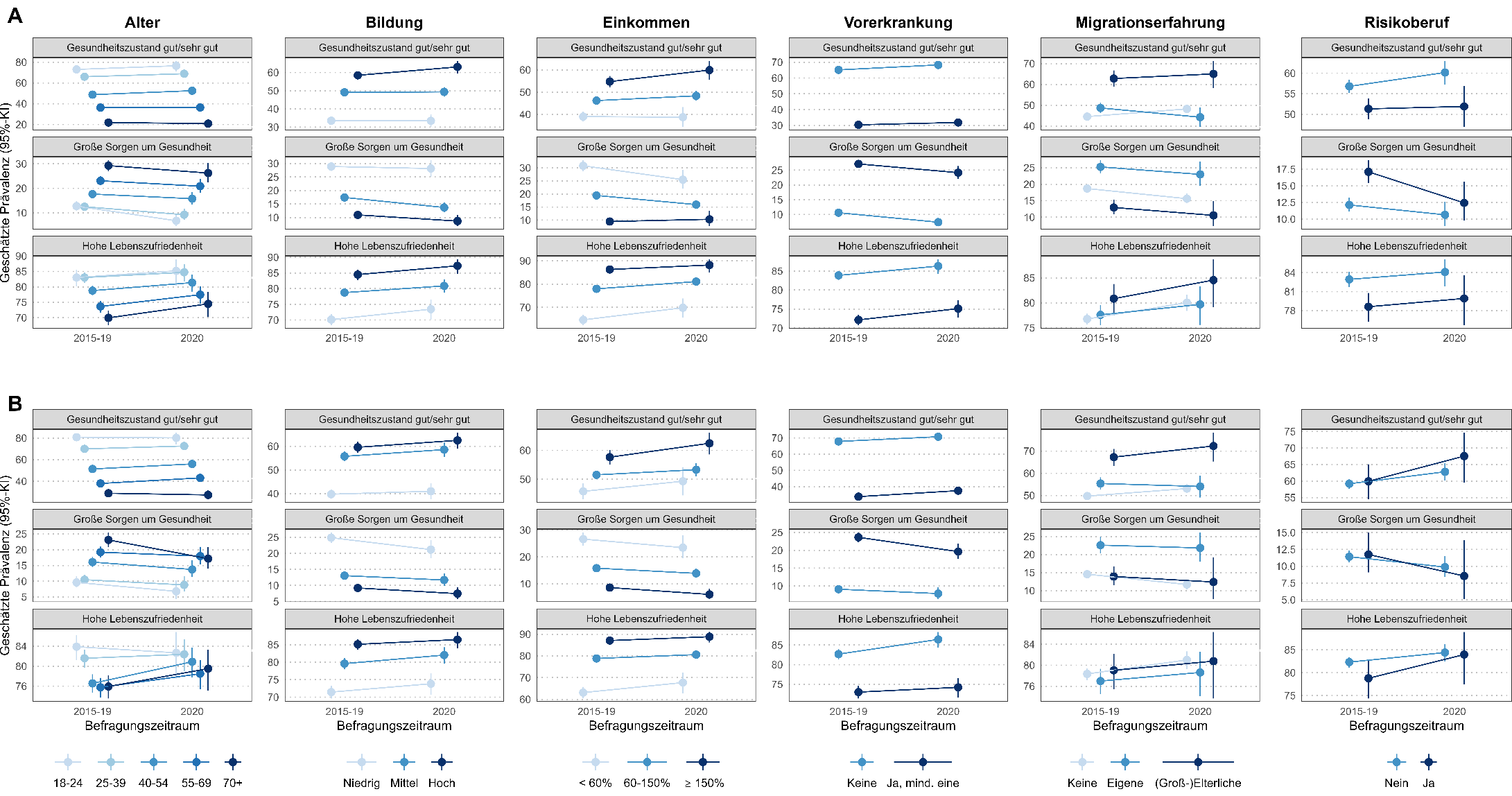


**Abbildung A1** Trends der subjektiven Gesundheit nach Stratifizierungsmerkmalen zwischen den Beobachtungszeiträumen 2015-19 und 2020 für Frauen (A) und Männer (B). Hinweis: Für eine pointierte Darstellung der Unterschiede variieren die Y-Achsen in der Skalierung (Quelle: eigene Abbildung).

1. Bundesagentur für Arbeit. Klassifikation der Berufe 2010 – überarbeitete Fassung 2020. Nürnberg: Bundesagentur für Arbeit, (2021). [↑](#footnote-ref-1)
2. In Anlehnung an Bauer A, Grienberger K, Matthes B, Jucknewitz R, Schramm A (2021) Berufe und Covid-19-Pandemie: Wie hoch ist das berufsspezifische Ansteckungsrisiko? In: IAB-Forum https://www.iab-forum.de/berufe-und-covid-19-pandemie-wie-hoch-ist-das-berufsspezifische-ansteckungsrisiko/. Zugegriffen: 07. März 2023 [↑](#footnote-ref-2)
